# Supplementary figures and images for: Evaluation of Parameters Which Influence Voluntary Ingestion of Supplements in Rats
Source: Animals (Basel). 2023 May 31;13(11):1827. doi: 10.3390/ani13111827 (PMC10251944; doi:10.3390/ani13111827)

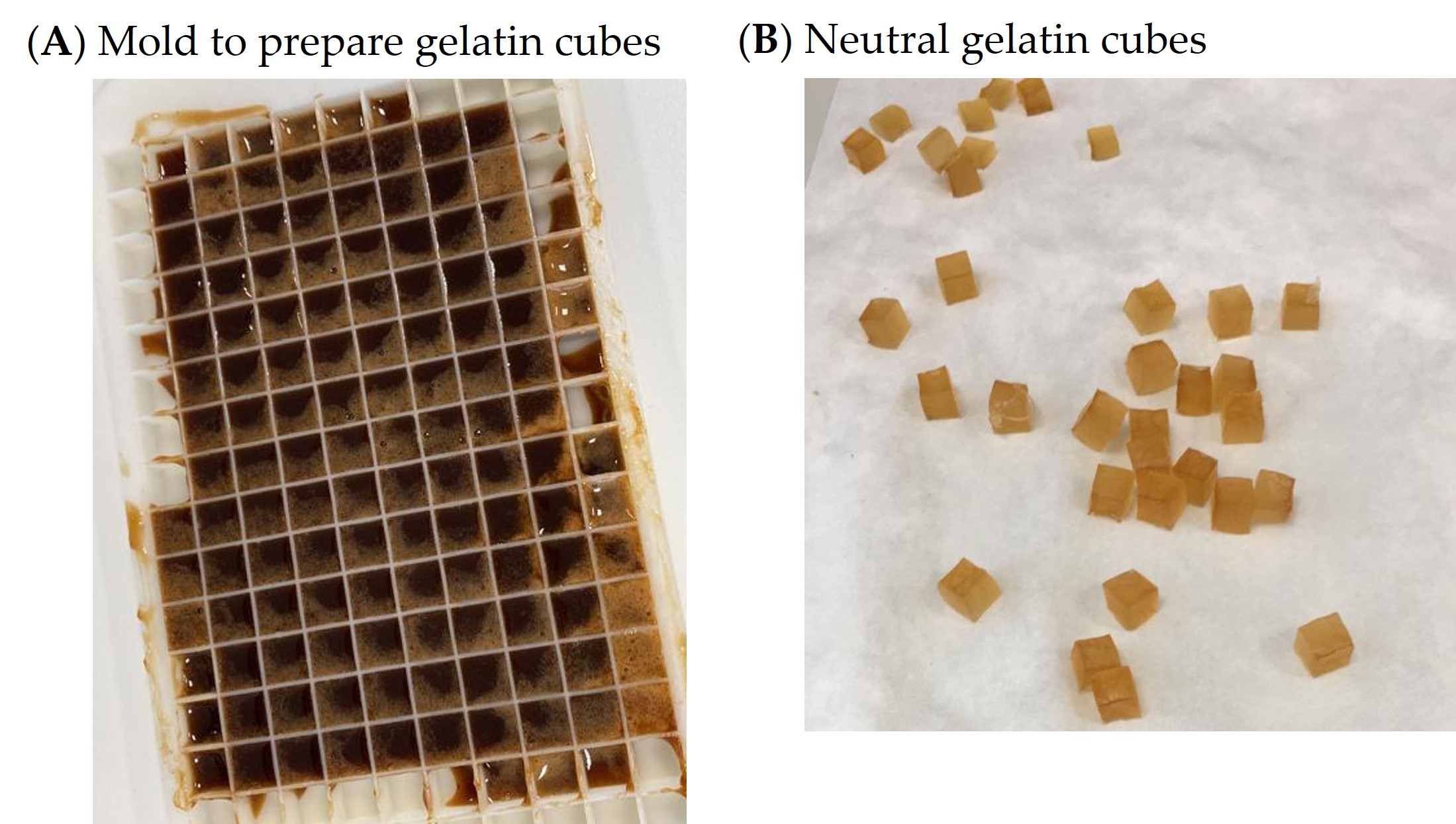

Supplement: Supplementary file 1 [file animals-13-01827-s001.zip › animals-2340747-supplementary-5.30/Figure S1_Preparation of gelatin cubes.jpg]
